# Normal weight diabetic patients versus obese diabetics: relation of overall and abdominal adiposity to vascular health

**DOI:** 10.1186/s12933-014-0141-8

**Published:** 2014-10-22

**Authors:** Alla Lukich, Dov Gavish, Marina Shargorodsky

**Affiliations:** Departments of Medicine, Wolfson Medical Center, Holon, Israel; Departments of Endocrinology, Wolfson Medical Center, POB 5, Holon, 58100 Israel; Sackler Faculty of Medicine, Tel Aviv University, Tel Aviv, Israel

## Abstract

**Objective:**

The present study investigated the impact of overall obesity defined by BMI and abdominal obesity defined by WC on vascular atherosclerotic changes in obese and normal weight diabetic subjects.

**Design and methods:**

285 subjects were divided according to presence diabetes mellitus (DM) and obesity: Group 1 included 144 nonobese subjects without DM; Group 2 consisted of 141 type 2 diabetic patients. Then diabetic patients were divided into two groups according to presence of overall obesity, defined by BMI and furthermore, abdominal obesity, defined by waist circumference (WC). Pulse wave velocity (PWV) and augmentation index (AI) were performed using SphygmoCor (version 7.1, AtCor Medical, Sydney, Australia).

**Results:**

*Between Group Comparisons by BMI*: Diabetic subjects with and without overall obesity did not differ from one another in terms of AI and PWV.

*Between Group Comparisons by WC*: AI as well as PWV increased consistently from Group 1 to Group 3, AI and PWV were significantly higher in abdominally obese diabetic subjects than in the diabetics without abdominal obesity (p = 0.008 and p = 0.013, respectively). Significant by-group differences in PWV and AI persisted after adjustment for age, sex, blood pressure, fasting glucose and BMI.

**Conclusions:**

Abdominal obesity defined by WC was associated with significantly higher AI and PWV in in both diabetic men and women; whereas overall obesity defined by BMI did not predict adverse vascular changes in this study population. Abdominal obesity was associated with an adverse effect on blood vessels, independently of age, sex, blood pressure, fasting glucose and BMI.

## Introduction

In diabetes, arterial stiffening is consistently observed across all age groups and may contribute, in part, to the excess cardiovascular morbidity and mortality observed in these patients [1,2]. Several pre-existing risk factors including hypertension, dyslipidemia and obesity may further increase the cardiovascular risk associated with hyperglycemia, leading to excess CVD risk at an earlier age. Physiopathology that links increased adiposity as well as diabetes to arterial stiffening is not precisely known. One suggested mechanism is through insulin resistance, which commonly accompanies obesity and type 2 diabetes mellitus. A reciprocal relationship exists between insulin resistance and endothelial dysfunction, considered to be a key initiating step in the atherosclerotic cascade [3,4]. However, not all type 2 diabetic individuals demonstrate similar insulin resistance as well as cardiovascular risk factor profiles. Obese type 2 diabetics have significantly decreased insulin-stimulated glucose disposal and insulin sensitivity index, confirming that insulin resistance is the major contributor to the pathogenesis of hyperglycemia in obese subjects with type 2 DM , whereas lean type 2 diabetics are characterized primarily by a defect in insulin secretion [5,6]. Currently, there is no consistent evidence regarding differences in term of cardiovascular morbidity and mortality between obese and nonobese diabetic patients. Even less information is available regarding vascular impact of different obesity anthropometric indexes such as waist circumference (WC) and body mass index (BMI) in obese and normal weight diabetic subjects.

Estimation of arterial stiffness has been reported to be a useful method for assessment of early preclinical atherosclerosis [7]. PWV, which is associated with higher risk of cardiovascular morbidity and mortality and AI, which recently been considered as a valid biomarker, provide valuable information regarding vascular health [8,9].

The present study was designed to investigate the impact of overall obesity defined by BMI and abdominal obesity defined by WC on vascular atherosclerotic changes in obese and normal weight diabetic subjects.

## Materials and methods

### Subjects

The study group consisted of 285 Caucasian subjects, (185 women and 100 men, mean age 61.4+/-10.9 years) who were recruited from the outpatient metabolic clinic and evaluated for the study. The study participants were divided into two groups according to presence of type 2 diabetes mellitus: 144 nonobese subjects without DM (DM-Ob-) defined as the control group and 141 diabetic subjects. Study participants were classified as diabetic if fasting plasma glucose level was ≥126 mg/dl on at least two blood samples, or if they were treated with antidiabetic medications. Then, diabetic patients were divided into two groups by presence of overall obesity, according to BMI: Group 2 consisted of 62 diabetic non obese subjects (DM + Ob-) and Group 3 contained 79 participants with diabetes and obesity (DM + Ob-). Obesity was defined using World Health Organization criteria (BMI > =30 kg/m^2^).

Additionally, diabetic subjects were divided into two groups according to presence of abdominal obesity. We classified male participants with WC >102 cm and female participants with WC >88 cm as subjects with abdominal obesity (AOb). Group 1 included 138 nondiabetic subjects without abdominal obesity (DM- AOb-) defined as the control group, Group 2 consisted of 67 diabetic subjects without abdominal obesity (DM- AOb-) and Group 3 contained 74 participants with diabetes and abdominal obesity (DM + AOb+). Patients included in the study had been stabilized according to their previous medical treatment in the outpatient clinic for up to three months before entrance to the study. This study had been approved by the local scientific committee, and all participants gave informed consent before entering the study.

### Biochemical parameters

Blood sampling for full chemistry and metabolic parameters, including total cholesterol, HDL and LDL cholesterol, triglycerides, fasting glucose, HbA1C, fasting insulin, CRP and serum aldosterone and plasma renin activity, was performed. Glucose was measured using the Aeroset chemistry system (Abbott Diagnostics), high-density lipoprotein cholesterol (HDL) and triglycerides were assayed using an Aeroset automated analyzer (Abbott Diagnostics, Berkshire, UK), low-density lipoprotein cholesterol (LDL) was calculated using Friedewald’s formula and insulin was measured using an immunometric assay specific for human insulin (Invitron, Monmouth, UK). Serum aldosterone and plasma renin activity were measured in a sitting position using commercially available radioimmunoassay. The lower limits of the serum aldosterone and plasma renin activity were 0.6 ng/dl and 0.1 ng/ml/h, respectively. The samples were measured in duplicate. Homeostasis model assessment-insulin resistance (HOMA-IR) was calculated by the following formula: fasting plasma insulin (mU/ml) × fasting plasma glucose (mg/dl)/405.

### Blood pressure and arterial stiffness measurement

The hemodynamic measurements were performed between 8 and 11 AM in a quiet room at 20–23°C after resting for 15 min. Blood pressure (BP) was measured using an automated digital oscillometric device (Omron model HEM 705-CP, Omron Corporation, Tokyo, Japan), and a mean of three readings was taken. Pulse wave velocity was measured by recording of the right carotid and the right radial artery pulse waveforms by two pressure transducers using the SphygmoCor Vx PWV System. The radial pressure waveform was recorded and subsequently transformed by using a validated generalized transfer function incorporated in the SphygmoCor (version 7.1, AtCor Medical, Sydney, Australia) to give an estimate of the corresponding central ascending aortic pulse wave. With the integral software, the central augmented pressure was calculated as the difference between the early and late systolic peaks of the estimated central pressure waveform. Central aortic augmentation index (AI) was calculated as the aortic augmented pressure expressed as a percentage of the pulse pressure and automatically adjusted to heart rate of 75 beats/minute. This technique, which has been validated for its reproducibility and used extensively, had been accepted as substantially equivalent to aortic pressure measured by invasive catheterization [10].

### Statistical analysis

Analysis of data was carried out using SPSS 11.0 statistical analysis software (SPSS Inc., Chicago, IL, USA). For continuous variables, such as hemodynamic, arterial compliance and chemistry parameters, descriptive statistics were calculated and reported as mean ± standard deviation. Normalcy of distribution of continuous variables was assessed using the Kolmogorov-Smirnov test (cut off at p = 0.01). Continuous variables were compared across groups using one-way analysis of variance (ANOVA). Variables for which across-group differences were detected underwent post hoc pairwise testing using the Bonferroni test. Categorical variables such as comorbidities and prescribed medications were described using frequency distributions and are presented as frequency (%). Categorical variables were compared across groups using the chi-square test (exact as needed). Pearson’s correlation analysis was used to calculate correlation coefficients to describe associations between continuous variables. Pulse wave velocity and augmentation index were modeled using multiple linear regression analysis with a backward, stepwise approach. For inclusion, the probability of F was set at 0.05, and at 0.10, for exclusion. Variables for inclusion were identified in univariate associations with the outcome of interest. All tests are two-sided and considered significant at p < 0.05.

## Results

### Between group comparisons by BMI

Demographic and clinical characteristics of the three groups are presented in Table 1. As expected, BMI was significantly higher in Group 3, as compared to Group 1 and Group 2; whereas, Groups 1 and 2 did not differ from one another. However, Group 1 and Group 2 differed significantly in terms of age, presence HTN, smoking and concomitant medications. As can be seen, parameters, such as total, LDL-, HDL- cholesterol, triglycerides, fasting glucose, HbA1C and HOMA-IR, differed significantly between Group 1 and Group 3. CRP levels and parameters of glucose homeostasis such as fasting glucose and HbA1C were significantly higher and HOMA-IR was marginally higher in obese diabetic subjects compared to non obese diabetics. Systolic as well as diastolic blood pressure values increased consistently from Group 1 to Group 3.

**Table 1:** **Demographic and clinical characteristics of study groups defined by BMI**

**Variables**	**Group 1**	**Group 2**	**Group 3**
	**DM-Ob-**	**DM + Ob-**	**DM + Ob+**
Age (years)	56.2 ±12.2	66.7 ±8.4*	61.4 ±8.7
BMI (kg/m2)	24.8 ±2.3	25.5 ±2.4	34.2 ±3.7*
Hypertension	10%	64.5%*	63.3%*
Smoker	8.3%	22.6%*	15.2%
Aspirin use	5.6%	53%*	39%*
Insulin use	0.0%	29%*	36.7%*
Metformin use	0.0%	80.6%*	69.6%*
ACEI/ARB use	16.0%	64.5%*	63.3%*
CC Blocker use	14.6%	38.7%*	32.9%
Beta blocker use	16.76%	48.4%*	46.8%*
Statin use	13.2%	69.4%*	58.2%*
Fasting plasma glucose(mg/dl)	93.4 ±9.8	147.9 ±43.9*	164.4 ±58.4*
HbA1C (%)	5.9 ±0.4	7.4 ±1.4*	8.1 ±1.7*
Low density lipoprotein cholesterol (mg/dl)	119.0 ±31.6	99.0 ±30.3	93.8 ±29.3*
High density lipoprotein cholesterol (mg/dl)	53.7 ±12.0	46.3 ±10.4*	45.1 ±13.1*
Triglycerides (mg/dl)	119.0 ±59.1	144.3 ±104.8	166.0 ±109.3*
C-reactive protein (mg/dl)	0.3 ±0.3	0.5 ±0.8*	0.9 ±1.3*
HOMA IR	1.9 ±1.2	6.0 ±13.0	10.9 ±17.7*
Creatinine (mg/dl)	0.9 ±1.2	1.1 ±0.9*	1.0 ±0.5*
Systolic blood pressure (mmHg)	124.7 ±15.6	141.4 ±19.9*	154.1 ±20.1*
Diastolic blood pressure (mmHg)	69.3 ±9.0	74.1 ±9.6*	78.7 ±9.9*
AI(%)	29.6 ±9.6	36.8 ±8.8*	35.6 ±11.3*
PWV (m/sec)	6.1 ±1.0	7.5 ±2.7*	7.5 ±3.2*

By-group comparisons made using one way analysis of variance (ANOVA) simultaneously comparing all three groups.

*p-value vs. group 1 (significant at the 0.05 level).

**Figure 1:**
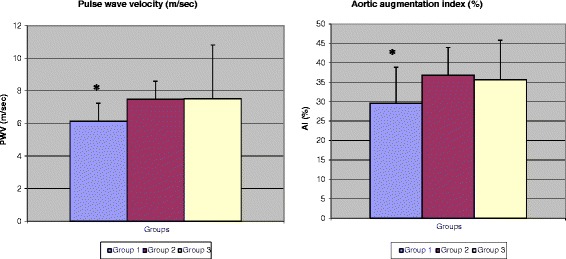
**AI and PWV parameters by overall obesity, according to BMI.**

As shown in Figure 1 and Table 1, diabetic subjects with and without overall obesity did not differ from one another in terms of AI and PWV (p = 0.064). As expected, non obese subjects without diabetes (DM-Ob-) had significantly lower PWV as well as AI than the two diabetic groups (p < 0.0001).

### Between group comparisons by WC

As can be seen in Table 2, WC was significantly higher in Group 3, as compared to Group 2 and Group 3; whereas, Groups 2 and 3 did not differ from one another. CRP level, fasting glucose, HbA1C and blood pressure increased consistently from Group 1 to Group 3.

**Table 2:** **Demographic and clinical characteristics of study groups defined by WC**

**Variables**	**Group 1**	**Group 2**	**Group 3**
	**DM-AOb-**	**DM + AOb-**	**DM + AOb+**
Age (years)	59.2 ±12.4	65.1 ±8.9*	62.0 ±8.7
BMI (kg/m2)	24.9 ±2.2	26.8 ±4.1	33.1 ±4.8*
Hypertension	10.9%	59.7%*	67.6%*
Smoker	8.7%	20.9%*	16.2%
Aspirin use	5.9%	49.3%*	41.9%*
Insulin use	0.0%	26.9%*	39.2%*
Metformin use	0.0%	74.6%*	74.3%*
ACEI/ARB use	16.7%	59.7%*	67.6%*
CC Blocker use	0.0%	35.8%*	36.8%*
Beta blocker use	15.2%	44.8%*	50%*
Statin use	13.8%	64.2%*	62.2%*
Fasting plasma glucose(mg/dl)	93.5 ±9.9	148.3 ±44.5*	166.0 ±57.6*
HbA1C (%)	5.9 ±0.4	7.5 ±1.6*	8.2 ±1.6*
Low density lipoprotein cholesterol (mg/dl)	119.8 ±31.1	89.1 ±27.6*	94.5 ±31.4*
High density lipoprotein cholesterol (mg/dl)	53.6 ±12.0	46.4 ±11.1*	44.6 ±12.4*
Triglycerides (mg/dl)	121.0 ±59.9	156.5 ±108.5*	156.6 ±105.2*
C-reactive protein (mg/dl)	0.3 ±0.3	0.5 ±0.5	0.9 ±1.5*
HOMA-IR	2.0 ±1.1	6.03 ±12.3*	10.8 ±17.8*
Creatinine (mg/dl)	0.9 ±0.2	1.0 ±0.4	1.1 ±0.7*
Systolic blood pressure (mmHg)	119.2 ±13.9	134.0 ±18.6*	153.3 ±20.6*
Diastolic blood pressure (mmHg)	67.2 ±8.6	73.1 ±9.7	78.4 ±10.3*
AI (%)	29.2 ±9.4	34.0 ±8.9*	38.8 ±10.4*
PWV(m/sec)	6.0 ±0.9	6.9 ±1.8*	7.9 ±3.5*

By-group comparisons made using one way analysis of variance (ANOVA).

simultaneously comparing all three groups.

*p-value vs. group 1 (significant at the 0.05 level).

**Figure 2:**
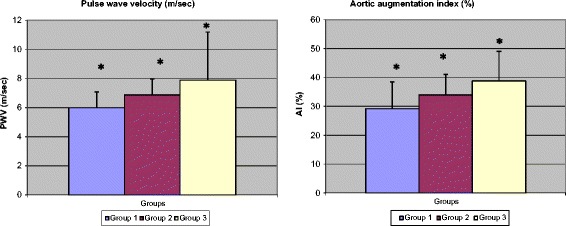
**AI and PWV parameters by abdominal obesity, according to WC.**

As shown in Figure 2 and Table 2, AI as well as PWV differed significantly between groups (p < 0.0001), such that both of them were significantly lower in non-obese non diabetic subjects than in the diabetic subjects with and without obesity. Groups 2 and 3 differed significantly from one another: AI and PWV were significantly higher in obese diabetic subjects than in the diabetic subjects without abdominal obesity (p = 0.008 and p = 0.013, respectively).

### General linear model of AI and PWV

Univariate GLM analysis was carried out to control for variables, differed significantly by groups (Table 3). This analysis was modeled using multiple linear regression analysis with a backward, stepwise approach. For inclusion, the probability of f was set at 0.05, and at 0.10, for exclusion. Included in the model of PWV were age, sex, blood pressure, fasting glucose and BMI. Additionally, group by WC was included as a fixed factor. The model of PWV was significant (p < 0.0001) and explained 14.5% of the variability in this outcome. Significant by-group differences in PWV persisted even after adjustment. BMI wasn’t the significant independent predictor of PWV.

**Table 3:** **Univariate GLM analysis for AI and PWV**

**Variables**	**AI**	**PWV**
	**Sig.**	**Sig.**
Age	0.000	0.813
Sex	0.003	0.007
Systolic BP	0.540	0.695
Fasting glucose	0.038	0.713
BMI	0.781	0.054
Group*	0.000	0.000

Multiple linear regression analysis was arrived at using a backward, stepwise approach with probability of F = 0.05 for entry and 0.15 for removal from the model.

*Group according to WC.

Additionally, general linear model of AI was carried out. The model was significant (p < 0.0001) and explained 30.8% of the variability in this outcome. Age, sex, blood pressure, fasting glucose and BMI were included as a covariate in this model. Significant by-group differences in AI persisted even after adjustment. The model itself was significant (p < 0.0001) and explained the variability of about 30.8% in AI.

## Discussion

In the present study, abdominal obesity defined by WC was associated with significantly higher AI and PWV in diabetic patients; whereas overall obesity defined by BMI did not predict adverse vascular changes in this study population. Abdominal obesity was associated with an adverse effect on blood vessels, independently of age, sex, blood pressure, fasting glucose and BMI. Combination of diabetes mellitus and abdominal obesity, but not overall obesity, was associated with significant deterioration in terms of arterial stiffness parameters.

Findings of the present study concur with recently published data demonstrated stronger association of abdominal obesity than BMI with total mortality among elderly subjects at high risk of cardiovascular disease, particularly among diabetic participants [11]. Additionally, it was shown that the measures of abdominal obesity correlates better than BMI with arterial stiffness evaluated by PWV, and with subclinical atherosclerosis evaluated by C-IMT, in healthy, diabetics and hypertensive subjects [12].

Previous clinical and experimental data show that abdominal adipose tissue can play an important role in the development of diabetes mellitus and it can also increase the risk of cardiovascular and all-cause mortality [13,14]. Body fat distribution is one of the major determinants of metabolic health, and visceral adiposity has a stronger correlation with metabolic abnormalities and cardiovascular disease than subcutaneous adipose tissue [15-17]. Visceral fat is metabolically active and is an important site for adipokines such as adiponectin and leptin, which plays an important role in insulin sensitivity, inflammation, lipid metabolism and atherogenesis. It was shown that low plasma adiponectin levels are significantly correlated with endothelial dysfunction, increased intima media thickness and progression of coronary artery calcification independently of other cardiovascular risk factors [18,19]. Leptin as well is involved in insulin sensitivity, angiogenesis, vascular and endothelial function [20]. Moreover, novel and traditional cardiovascular risk factors such as asymmetric dimethyl-arginine ADMA levels and LDL-cholesterol are strongly associated with increased arterial stiffness among pre-diabetic subjects [21].

It has been demonstrated that excess body fat, abdominal visceral fat, and larger waist circumference have been associated with accelerated arterial stiffening independently of blood pressure levels, ethnicity and age in older adults [22]. Moreover, both WC and sagittal abdominal diameter (SAD) are associated with subclinical organ damage such as PWV and carotid intima-media thickness and provided information on inflammation, atherosclerosis and arterial stiffness in type 2 diabetic patients [23]. However, it has also been reported that SAD was more independent in predicting arterial stiffness over time, compared with WC, in middle-aged men and women with type 2 diabetes [24], Recently , it has been demonstrated that neck circumference is associated with an increased PWV in hypertensive adults, independent of other metabolic risk factors [25].

The pathophysiology that links abdominal adiposity to arterial stiffening is not precisely known. One suggested mechanism is through insulin resistance, which commonly accompanies obesity. A reciprocal relationship exists between insulin resistance and endothelial dysfunction, considered to be a key initiating step in the atherosclerotic cascade. Reduced insulin action in peripheral tissue impairs endothelium-dependent vasodilatation [3] and increases the local activity of a variety of growth factors in vascular tissue [26,27], promoting collagen production and the development of vascular smooth muscle cell (VSMC) hypertrophy [28]. It has been shown that obese young adults who both lower their insulin levels and lose weight showed the greatest improvement in vascular stiffness [29]. In addition, the pro-inflammatory state typical of obesity stimulates reactive oxygen species production, inhibits nitric oxide production by reducing levels of NO synthase in vascular smooth muscle cells and endothelial cells and promotes endothelial apoptosis [30,31].

In concurrence with previous studies, the present study did not detect the gender-related differences in the association between abdominal obesity measures and arterial stiffness in diabetic men and women. However, it has also been reported, that increasing BMI, WC, visceral fat area and fat mass were independently associated with higher PWV in women, but not in men, after adjustment for age, hypertension and type 2 diabetes [32].

The individuals with normal weight and increased levels of abdominal obesity could be genetically predisposed to the development of diabetes mellitus as well as CV disease. Since vascular changes inflicted by multiple environmental and genetic factors, develop years before an event, detection of vascular damage can serve as a predictor of future cardiovascular complications. In the present study, WC was a significant independent predictor of early vascular adverse changes detected by using PWV and AI. Therefore, abdominal obesity defined by WC, might be a better predictor of arterial stiffness as well as future cardiovascular events in diabetic patients.

In conclusion, the present study demonstrated that abdominal obesity, defined by WC is associated with an adverse effect on blood vessels, independently of age, sex, and blood pressure, parameters of glucose homeostasis and BMI in type 2 diabetic patients. Combination of diabetes mellitus and increased WC, but not increased BMI, was associated with significant deterioration in terms of arterial stiffness parameters in this study population. The precise mechanisms for these vascular changes, as well as overall clinical impact of WC reduction on cardiovascular outcomes deserve further investigation.
